# Dr. Isaiah Forbes

**Published:** 1885-09

**Authors:** 


### Obituary.

---

DR. ISAIAH FORBES.—Dr. Isaiah Forbes, who died at St. Louis, July 15th, 1885, at the age of 75 years, will long be remembered in the west as one of the leading dentists of his day.

He began the practice of his profession in St. Louis in 1837, when the city contained only from eight to ten thousand people. Having studied dentistry in the office of Dr. Ambler of New York, he sought the West as the place most likely to open for him an avenue of useful labor. In this he was not disappointed. He early gained the confidence of the people and retained it through a period longer than any other man that ever practiced in this city. Dr. Forbes was not what could be called a brilliant man in his profession. He had no special aptitude to any one particular branch. He was not a close investigator. Yet he was endowed with that faculty of encouraging everything that pointed to a broad and liberal professional advancement. It is not always so much in what a man accomplishes himself as it is in what he helps others to, that we find the secret of advancement. Dr.

Forbes' open, generous face indicated the patron of, rather than the plodder after, scientific truths.

Tall, erect, with a clear, open countenance, elastic step, and infinite grace and dignity, he was the peer of any gentleman in the land.

His standing in society was particularly fortunate. He occupied a position rarely accorded to the dental practitioner. For twelve years he officiated as member of the school board, and for a long time was a prominent worker in the historical society of the city.

His name was never mentioned in connection with any public or charitable enterprise without carrying with it the weight of a leading citizen.

He was not one of the kind that always lived inside of a decayed tooth. While he was proud of his profession he yet had sufficient good sense and respect for the universal fitness of a thing to leave it at home when he moved abroad among his fellow men. J. C.

ST. LOUIS, July 21st, 1885. —*Items of Interest.*

---

